# Increase in the rate of azithromycin-resistant *Streptococcus pneumoniae* isolates carrying the *erm(B)* and *mef(A*) genes in Taiwan, 2006–2010

**DOI:** 10.1186/s12879-014-0704-z

**Published:** 2014-12-19

**Authors:** Dodi Safari, Lu-Cheng Kuo, Yu-Tsung Huang, Chun-Hsing Liao, Wang-Huei Sheng, Po-Ren Hsueh

**Affiliations:** Eijkman Institute for Molecular Biology, Jakarta, Indonesia; Department of Internal Medicine, National Taiwan University Hospital, National Taiwan University College of Medicine, Taipei, Taiwan; Department of Internal Medicine, Far Eastern Memorial Hospital, Taipei, Taiwan; Department of Laboratory Medicine, National Taiwan University Hospital, National Taiwan University College of Medicine, Taipei, Taiwan

**Keywords:** Streptococcus pneumoniae, Azithromycin-resistant, Tigecycline In Vitro Surveillance in Taiwan (TIST) study

## Abstract

**Background:**

This study investigated the molecular characteristics of azithromycin-resistant *Streptococcus pneumoniae* in Taiwan.

**Methods:**

A total of 486 non-duplicate isolates of azithromycin-resistant *S. pneumoniae* recovered from various clinical sources of patients treated at 22 different hospitals in Taiwan from 2006 to 2010. The presence of *erm*(B) and *mef*(A) genes using duplex PCR, multilocus sequence typing (MLST), and pulsed-field gel electrophoresis of these isolates were studied.

**Results:**

Of the isolates tested, 59% carried the *erm(B)* gene, 22% carried the *mef(A)* gene, and 19% carried both genes. The prevalence of isolates carrying the *erm(B)* and *mef(A) genes* increased from 10% (11/110) in 2006 to 25% (15/60) in 2010 (*p*-value = 0.0136). The majority of isolates carrying both *erm(B)* and *mef(A)* genes belonged to serotypes 19 F (64%) followed by 19 F A (24%). Of these isolates, 33% were sequence type 320 (ST320), 32% were ST236, and 12% were ST271.

**Conclusions:**

The increase in incidence of *mef(A)/erm(B)*-positive azithromycin-resistant *S. pneumoniae* isolates during the study period was primarily due to serotypes 19 F and 19A and ST236 and ST320.

**Electronic supplementary material:**

The online version of this article (doi:10.1186/s12879-014-0704-z) contains supplementary material, which is available to authorized users.

## Background

*Streptococcus pneumoniae* is a leading cause of bacterial pneumonia, meningitis, and sepsis worldwide. Since 1965, many cases of infections due to drug-resistant *S. pneumoniae* have been reported [1]. The emergence of antimicrobial resistance is correlated with selective pressure from the use, often inappropriate, of antimicrobial agents and results in increased mortality, morbidity, and health care costs [2]. Antibacterial resistance in *S. pneumoniae* is increasing, affecting principally β-lactams and macrolides (azithromycin, erythromycin, or clarithromycin) with prevalence ranging between 1% and 90% depending on the geographical area [3]. Fluoroquinolone resistance has also been reported in countries with high levels of antibacterial resistance and consumption [3].

Macrolide resistance in *S. pneumoniae* is most often mediated by two mechanisms: target-site modification encoded by the *erm*(B) gene and active drug efflux mediated by a membrane efflux pump encoded by *mef*-class genes [4]. Song *et al.* reported the *erm(B)* gene was found in >50% of pneumococcal isolates either alone or in combination with *mef(A)* among *S. pneumoniae* isolates from 10 Asian countries during 1998–2001 [5]. In Finland and Germany, the most frequent macrolide resistance determinant carried was the *mef* gene [6],[7]. Macrolide resistance among pneumococcal isolates in Alaska recovered from 1986–2010 was also reported to be predominantly mediated by *mef* genes and this has not changed significantly over time [8]. However, the authors of the study reported a significant increase in the proportion of isolates that possess both *erm(B)* and *mef(A),* primarily among serotype 19A isolates.

Bowers *et al.* reported that of 592 clinical pneumococcal isolates collected in Arizona from 1999 to 2008, all isolates carrying the erythromycin-resistant genes *mef(E) and erm(B)* were multidrug-resistant clonal lineages of Taiwan 19 F-14 and most were multilocus sequence type (ST) 320 [9]. In China, recent studies have shown that erythromycin-resistant isolates commonly carry both genes and that the majority of isolates belong to ST271, ST320, ST236, with clonal complex 271 (CC271) being the most frequently isolated CC [10]-[12]. In 2005, two predominant macrolide-resistant *S. pneumoniae* CCs, namely CC271 and CC15, were identified in New South Wales, Australia [13]. Recently, Tsai *et al.* reported the prevalence of serotype 19A pneumococcal isolates increased significantly in Taiwan from 2006 to 2010 and that more than 90% of the isolates were non-susceptible to azithromycin [14]. In the current study, we investigated the molecular characteristics of azithromycin-resistant *S. pneumoniae* recovered from various clinical sources of patients who were treated at 22 different hospitals in Taiwan from 2006 to 2010.

## Methods

### Bacterial isolates

A total of 530 consecutive and non-duplicate pneumococcal isolates were collected from various clinical specimens of patients treated at 22 different hospitals in Taiwan during a 3-month period per year, with a maximum number of isolates per year of 10 during 2006–2008 and 5 during 2009–2010 [14]. Among these isolates, 486 were not susceptible to azithromycin [14]. These pneumococcal isolates were collected as part of the Tigecycline *In Vitro* Surveillance in Taiwan (TIST) study, a nationwide, multicenter, prospective surveillance study conducted in 12 regional hospitals (500–1000 beds) and 10 medical centers (1200–3000 beds) (eight in northern, four in central, six in southern and two in eastern Taiwan) from January 2006 to December 2010 [15], Pneumococcal isolates were identified at each hospital and the identification was confirmed by the central laboratory at the National Taiwan University Hospital [15]. Serotype determination by a latex agglutination method and antimicrobial susceptibility testing by the broth microdilution method were performed as described previously [14]. Isolates were collected as part of standard patient care and no ethical approval required for your use.

### Detection of *erm*(B) and *mef*(A) genes

The detection of *erm*(B) and *mef*(A) was performed by duplex PCR as previously described [5],[16].

### Pulsed-field gel electrophoresis (PFGE) analysis

PFGE analysis of isolates was performed as described previously [17],[18]. The Dice coefficient of similarity was calculated and the unweighted pair group method with arithmetic averages (UPGMA) was used for cluster analysis. Isolates with coefficients of similarity ≥80% were considered to be the same cluster [18].

### Multilocus sequence typing (MLST)

MLST was performed as described previously [19]. Allele profiles and sequence types were determined using the MLST database (http://spneumoniae.mlst.net/).

### Statistical analysis

Statistical analyses were conducted using GraphPad Prism V5.0 (GraphPad Software, San Diego, CA, USA).

## Results

### Prevalence of isolates carrying the *erm(B)* and *mef(A)*genes

Among the 486 isolates, 59% carried the *erm(B)* gene*,* 22% carried the *mef(A)* gene, and 19% carried both genes (Table 1). The prevalence of isolates carrying the *erm(B)* gene did not differ significantly from year to year (*p*-value = 0.2436) (Table 1); the prevalence of isolates carrying the *mef(A*) gene declined significantly from 30% in 2006 to 5% in 2010 (*p*-value = 0.0001); and the prevalence of isolates carrying both genes increased significantly from 10% in 2006 to 25% in 2010 (*p*-value = 0.0136) (Table 1). There were no obvious geographic differences with respect to the distribution of isolates carrying *erm(B), mef(A)*, or both genes (data not shown).

**Table 1:** **Prevalence of** ***erm(B)*** **and** ***mef(A)*** **genes among azithromycin-resistant** ***Streptococcus pneumoniae*** **isolates from 22 hospitals in Taiwan from 2006 to 2010**

Resistant gene	No. (%) of isolates, by study period					Total (n = 486)
	2006	2007	2008	2009	2010	
	(n = 110)	(n = 110)	(n = 153)	(n = 53)	(n = 60)	
*erm(B)*-positive	66 (60)	65 (59)	92 (60)	24 (45)	42 (70)	289 (59)
*mef(A)*-positive	33 (30)	26 (24)	33 (22)	11 (21)	3 (5)	106 (22)
*mef(A)/erm(B)*-positive	11 (10)	19 (17)	28 (18)	18 (34)	15 (25)	91 (19)
*P*-value*	-	0.1683	0.8716	0.0224	0.3091	

*P-value for temporal change of *mef(A)/erm(B)-*positive by study period.

### Serotype and sequence type of isolates carrying both *erm(B)* and *mef(A*) genes

All isolates of serotype 3 and 15B carried only *erm(B)* (Table 2). The majority of the other main serotypes also carried only the *erm(B)* gene, namely serotype 23 F (73%), 14 (87%), 23A (86%), and 6B (54%) (Table 2). Among serotype 19 F isolates, 13% carried the *erm(B)* gene, 36% carried the *mef(A)* gene, and 52% carried both genes. The majority of serotype 19A isolates carried both *erm(B)* and *mef(A)* genes (61%) (Table 2). Of 91 these isolates carried both genes, 58 (64%) of isolates were belong to serotype 19 F, followed by 19A (22/91; 24%).

**Table 2:** **Correlation between the main serotypes of azithromycin-resistant** ***Streptococcus pneumoniae*** **isolates and macrolide-resistant genes**

Serotype*	No. of isolates	No. (%) of isolates, by resistant gene		
		*erm(B)* -positive	*mef(A)* -positive	*mef(A)/erm(B)* -positive
19F	112	14 (13)	40 (36)	58 (52)
23 F	90	66 (73)	21 (23)	3 (3)
14	71	62 (87)	8 (11)	1 (1)
6B	66	35 (53)	29 (44)	2 (3)
19A	36	10 (28)	4 (11)	22 (61)
3	23	23 (100)	0 (0)	0 (0)
15B	15	15 (100)	0 (0)	0 (0)
23A	16	14 (88)	1 (6)	1 (6)
NT	28	25 (89)	3 (11)	0 (0)
Others**	29	25 (86)	0 (0)	4 (14)

NT: nontypeable.

*Data of serotype were used in this study as reported by Tsai HY et al. [14],

**Include 9 V (n = 9), 6A (n = 9), 10A (n = 2), 20 (n = 1), 11A (n = 1), 15A (n = 5), 22 F (n = 2).

The distribution of isolates harboring *erm(B)* and *mef(A)* genes by sequence type was 33% for ST320, 32% for ST236, 12% for ST271, 8% for ST81, 2% for ST283 and ST8525, and 11% for other sequence types (Table 3). The majority of ST236 (28/29) and ST271 (10/11) clones belonged to serotype 19 F. Isolates of clone ST320 mainly belonged to serotype 19 F (16/30) and serotype 19A (13/30) (Table 3). Seven isolates of clone ST81 were identified as belonging to serotype 23 F (n = 2), 23A (n = 1) and 6A (n = 4). Based on the results of MLST allelic profiling, the ST236 and ST81 clones were identified as reference strain of PMEN global clone Taiwan^19F^-14 and reference strain of PMEN global clone Spain^23F^-1 respectively (Table 3). ST320 and ST271 clones were identified as a double-locus variant (DLV) and a single-locus variant (SLV) of the worldwide-established Taiwan^19F^-14 (ST236) clone respectively.

**Table 3:** **Sequence type and Serotype of azithromycin-resistant** ***Streptococcus pneumoniae*** **isolates with PCR positive for** ***erm(B)*** **/** ***mef(A)*** **genes**

ST	No. (%) of isolates	Serotype (no. of isolates)	Related PMEN clone [ [20]] ^**^
236	29 (32)	19 F (28), 19A (1)	Taiwan^19F^-14/ST236
320	30 (33)	19F (16), 19A (13), 14 (1)	DLV of Taiwan^19F^-14/ST236
271	11 (12)	19 F (10), 19A (1)	SLV of Taiwan^19F^-14/ST236
81	7 (8)	23 F (2), 23A (1), 6A (4)	Spain^23F^-1/ST81
283	2 (2)	19 F (2)	-
8525	2 (2)	19 F (2)	-
Others^*^	10 (11)	19 F (4), 19A (3), 23 F (1), 6B (2)	-

ST: Sequence type; PMEN: Pneumococcal molecular epidemiology network.

^*^Others (n): ST3111 (1), ST3164 (1), ST6993 (1), ST7123 (1), ST237 (1), ST257 (1), ST3625 (1), ST2993 (1), ST76 (1), new ST (1); DLV, double locus variant; SLV, single-locus variant;

^**^PMEN website. Available: http://web1.sph.emory.edu/PMEN/pmen_table2.html.

### Clusters of isolates carrying both *erm(B)* and *mef(A)*genes

**Figure 1:**
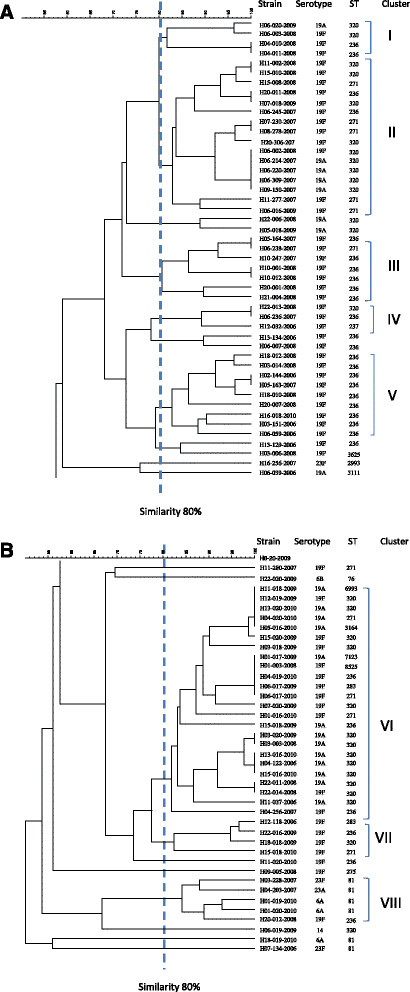
**A phylogenetic tree analysis based on pulsed-field gel electrophoresis profiles with** ***Sma*** **I among isolates of azithromycin-resistant** ***Streptococcus pneumoniae*** **carrying both** ***erm(B)/mef(A)*** **genes.**

We constructed a phylogenetic tree based on PFGE profiles and found no specific clustering for the strains of serotype 19 F and 19A or for the three major sequence types (ST320, ST271, and ST236) (Figure 1). In this study, the isolates carrying both *erm(B)* and *mef(A)* were stratified into eight clusters (Cluster I to VIII) by PFGE (Figure 1 and Table 4). Clusters III, IV, V, and VII corresponded to the isolates with serotype 19 F (Table 4). Isolates belonging to the same cluster can have different serotypes and STs. Furthermore, several isolates with the same ST also exhibited different serotypes and pulsotypes. The majority of isolates of serotype 19A were in cluster VI (13/24, 54%). ST320 clone isolates belonged to cluster VI (13/24, 54%), cluster II (9/16, 56%), and cluster I (2/4, 50%). Meanwhile, isolates of clone ST236 were frequently clustered in cluster V (9/9, 100%) and III (6/7, 86%) (Table 4). The majority of ST81 clone isolates were clustered in cluster VIII (4/5, 80%).

**Table 4:** **Pulsed-field gel electrophoresis (PFGE) clusters, serotypes and sequence types of azithromycin-resistant** ***Streptococcus pneumoniae*** **isolates with PCR positive for** ***erm(B)*** **/** ***mef(A)-*** **positive genes**

PFGE cluster*	No. of isolates (n = 87)**	Serotype (no. of isolates)	Sequence type (no. of isolates)
I	4	19 F (3), 19A (1)	320 (2), 236 (2)
II	16	19 F (12), 19A (4)	320 (9), 236 (2), 271 (5)
III	7	19 F (7)	236 (6), 271 (1)
IV	3	19 F (3)	320 (1), 236 (1), 237 (1)
V	9	19 F (9)	236 (9),
VI	24	19 F (11), 19A (13)	320 (13), 236 (3), 271 (3), 6993 (1) 3164 (1), 7123 (1), 8525 (1), 283 (1),
VII	4	19 F (4)	320 (1), 236 (1), 271 (1), 283 (1)
VIII	5	19 F (1), 6A (2), 23 F (1), 23A (1),	236 (1), 81 (4)
Un-clustered	15	19 F (7), 19A (3), 23 F (2), 6B (1), 6A (1), 14 (1)	320 (3), 236 (4), 271 (1), 81 (2), 76 (1), 275 (1), 2993 (1), 3111 (1), 3625 (1)

^*^PFGE-based clusters were defined as groups of 3 or more isolates with ≥80% similarity on the dendrogram.

**PFGE data were not available for four isolates (H03-020-2010, H04-004-2008, H12-017-2009, H15-012-2008).

## Discussion

Azithromycin is the most commonly used macrolide in the treatment of community-acquired pneumonia and other respiratory tract infections in Taiwan. The rate of susceptibility to azithromycin remained stationary from 2006 to 2010 in Taiwan, although the numbers of isolates randomly collected in 2009 and 2010 were lower than in 2006 to 2008 [14]. In Taiwan, PCV-7 vaccination was introduced in October 2005 and PCV-13 was introduced in July 2010. Nevertheless, some studies have shown that changes in antimicrobial susceptibility before and after implementation of the PCV-7 vaccine were not associated with serotypes [14]. Our finding of increase in the rate of azithromycin-resistant *S. pneumoniae* isolates carrying the *erm*(B) and *mef*(A) genes from from 10% in 2006 to 25% in 2010 after the introduction of the pneumococcal conjugate vaccine in Taiwan. These findings are in line with a previously published report on the PROTEKT US surveillance study from 10% in 2000 1 to 16% in 2003 [21], the study in Alaska from 0% in 1986 to 21% in 2010 [8], and the study in Canada from 3% in 1998 to 19% in 2008 [22].

In this study, the majority of azithromycin-resistant isolates carrying both *mef(A)* and *erm(B)* genes was serotype 19 F (58/91; 64%), followed by19A (22/91; 24%) and is similar to a previous published report in Korea, 57% of carried both genes were serotype 19 F (44/77, 57%), followed by 19A (21/77, 30%)7.5%) [23]. However, the study in Alaska showed 79% of isolates carrying both genes was serotype 19A (15/19), followed by 19 F (3/19; 16%) [8].

We investigated further via MLST and PFGE all isolates carried both the *erm(B)* and *mef(A)*, and identified 33% of these to be of ST320, followed by ST236 (32%), ST271 (12%), and other STs (23%). Previously, it was reported in Taiwan that the CCs related to Spain^23F^-1, Taiwan^19F^-14, and Taiwan^23F^-15 were responsible for the spread of isolates with high-beta-lactam resistance [24],[25]. Recently, the *S. pneumoniae* serotype 19A ST320 clone, derived from an international Taiwan^19F^-14 (ST236) clone, has become prevalent in many countries, including Taiwan [25]. In Arizona, the isolates carrying both *mef(E)/erm(B)*-positive genes are multidrug-resistant clonal lineages of Taiwan^19F^-14 [9].

In the last two decades, PFGE and MLST have become the main genotyping methods for assessing the genetic diversity of isolates [26]. Although both methods are time- and labor-consuming, they are useful for studying the local and global epidemiology of *S. pneumoniae*. In the present study, discrepancies of typing results by these two methods occurred. Since pneumococci are capable of undergoing capsular switching and are recognized as one of the most recombinogenic bacteria, additional typing methods, i.e. multiple-locus variable number tandem repeat analysis and MILST, have been developed recently to offer better discrimination in *S. pneumoniae* isolates [26].

## Conclusions

The increase in incidence of *mef(A)-* and *erm(B)*-positive azithromycin-resistant *S. pneumoniae* isolates during the study period was primarily due to serotypes 19 F and 19A and ST236 and ST320.

## Authors’ original submitted files for images

Below are the links to the authors’ original submitted files for images. Authors’ original file for figure 1

## Abbreviations

MLST: Multilocus sequence typing
TIST: Tigecycline In Vitro Surveillance in Taiwan
PCR: Polymerase chain reaction
ST: Sequence type
CC: Clonal complex
PFGE: Pulsed-field gel electrophoresis
UPGMA: Unweighted pair group method with arithmetic averages
PMEN: Pneumococcal molecular epidemiology network
DLV: Double-locus variant
SLV: Single-locus variant
