# Supplementary figures and images for: Increase in the rate of azithromycin-resistant Streptococcus pneumoniae isolates carrying the erm(B) and mef(A) genes in Taiwan, 2006–2010
Source: BMC Infect Dis. 2014 Dec 19;14:704. doi: 10.1186/s12879-014-0704-z (PMC4279982; doi:10.1186/s12879-014-0704-z)

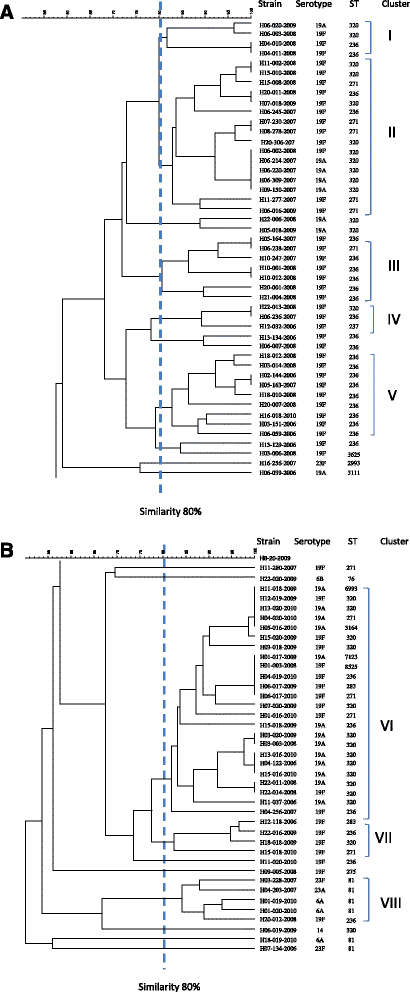

Supplement: Supplementary file 1 — Authors’ original file for figure 1 [file 12879_2014_704_MOESM1_ESM.gif]
